# Probing the Electron Capture Dissociation Mass Spectrometry of Phosphopeptides with Traveling Wave Ion Mobility Spectrometry and Molecular Dynamics Simulations

**DOI:** 10.1007/s13361-015-1094-1

**Published:** 2015-04-02

**Authors:** Doyong Kim, Pei-Jing Pai, Andrew J. Creese, Andrew W. Jones, David H. Russell, Helen J. Cooper

**Affiliations:** 1 Department of Chemistry, Texas A&M University, College Station, TX 77840 USA; 2 School of Biosciences, University of Birmingham, Edgbaston, Birmingham, B15 2TT UK

**Keywords:** ECD, TWIMS, MDS, Phosphopeptides

## Abstract

Electron capture dissociation mass spectrometry offers several advantages for the analysis of peptides, most notably that backbone c and z fragments typically retain labile modifications such as phosphorylation. We have shown previously that, in some cases, the presence of phosphorylation has a deleterious effect on peptide sequence coverage, and hypothesized that intramolecular interactions involving the phosphate group were preventing separation of backbone fragments. In the present work, we seek to rationalize the observed ECD behavior through a combination of ECD of model peptides, traveling wave ion mobility mass spectrometry and molecular dynamics simulations. The results suggest that for doubly protonated ions of phosphopeptide APLpSFRGSLPKSYVK a salt-bridge structure is favored, whereas for the doubly-protonated ions of APLSFRGSLPKpSYVK ionic hydrogen bonds predominate.

**Electronic supplementary material:**

The online version of this article (doi:10.1007/s13361-015-1094-1) contains supplementary material, which is available to authorized users.

**Graphical Abstract:**
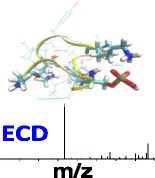
ᅟ

## Introduction

Electron capture dissociation (ECD) is a tandem mass spectrometry technique in which trapped ions are irradiated with low energy electrons [1, 2]. ECD has proven to be highly useful in the analysis of peptides and proteins, largely thanks to the retention of labile post-translational modifications, such as phosphorylation, on the backbone *c* and *z* fragments [3–6]. The approach has been applied to the large-scale analysis of phosphoproteins [7].

In earlier work, we demonstrated that the presence of phosphorylation can have a deleterious effect on the ECD fragmentation of doubly-protonated peptide ions [8]. For peptides with sequence APLSFRGSLPKSYVK, where each of the serines is variably phosphorylated, ECD of the doubly charged precursor revealed no ECD fragments between the phosphoserine and either the lysine residue at position 11 or the arginine at position 6. We concluded that noncovalent interactions between the basic side chains and the phosphoserine were preventing separation of any ECD fragments that had formed. (The retention of noncovalent interactions following electron capture is well established [9–11]). Support for this hypothesis is found in the work of Ruotolo et al. [12, 13] and Thalassinos et al. [14]. These researchers showed that collision cross sections (CCS) for some phosphopeptide ions differ from those for their unmodified counterparts. Each of the studies reported negative deviations for the phosphopeptides (i.e., CCS that fall below the random coil trend line) This behavior suggests compaction of the conformation owing to intramolecular interactions between the phosphate group and protonated side chains of arginine or lysine.

Here, we probe the nature of these noncovalent interactions, either salt-bridge or ionic hydrogen bond, by use of a combination of traveling wave ion mobility spectrometry and molecular dynamics simulations. Salt-bridges are electrostatic interactions between amino acid residues of opposing charge and play important roles in protein stability [15, 16]. Iakoucheva et al. have noted that basic residues, lysine and arginine, are found at high frequency in protein sequences near phosphorylation sites, suggesting that salt-bridges are important in phosphoprotein structures [17]. It might be expected that any solution-phase salt-bridge structures are destroyed as a result of proton transfer on transition to the gas phase [18]. The question of whether solution-phase salt-bridges are retained in the gas phase has been addressed by a number of researchers. Williams and co-workers showed the most stable form of singly protonated peptide ions of bradykinin is the salt-bridge structure [19]. Further work by that group [20] revealed that for dipeptides of sequence Xxx-Arg (Xxx is Gly, Val, Pro, Lys, His, or Arg), the ArgArg peptide ion has a salt-bridge structure, whereas the remaining dipeptide ions have a single formal charge site, suggesting that the presence of multiple basic residues stabilizes the salt-bridge structure. More recently, one of our groups applied cryogenic ion mobility-mass spectrometry to demonstrate that the presence of solution-phase salt-bridges aids retention of solution-phase structure in the gas phase [21].

ECD and the related technique of electron transfer dissociation have previously been applied to the investigation of intra- and intermolecular electrostatic interactions in the gas-phase structure of peptides and proteins. Breuker et al. applied ECD to the study of gas-phase structure of the protein KIX [22], revealing that salt-bridges and ionic hydrogen bonds conferred stability, and to the gas-phase unfolding of ubiquitin [23], again finding stabilization by salt-bridges. Vachet and co-workers showed, via ETD experiments, that many known solution-phase salt-bridges were retained in the gas-phase structures of protein ubiquitin, CRABP1, azurin, and β-2-microglobulin [24]. Woods and co-workers have demonstrated the gas-phase stability of the electrostatic interaction between arginine and phosphoserine in noncovalent complexes [25], and used ECD and ETD to identify the sites of interaction [26]. The presence of phosphopeptide zwitterions has also been demonstrated by Hakansson and co-workers in their work on negative ion ECD [27].

The relationship between phosphopeptide structure and ECD behavior was probed by Tureček and co-workers [28] in their comparison of ECD, ETD, and ECID (electron capture induced dissociation) of doubly protonated phosphopeptides pSAAAR, ApSAAR, AApSAR, and AAApSR. They used a combination of molecular dynamics simulations and DFT calculations to obtain structures of the lowest energy conformers to explain the experimentally observed aberrant ECD fragmentation. These phosphopeptides are simple enough to permit DFT calculations but complex enough that intramolecular interactions between side chains are anticipated. In further work [29], they compared theoretical calculations of structure with results from drift tube ion mobility spectrometry and traveling wave ion mobility (and IR action spectroscopy). They found broad agreement between theory and experiment, but could not directly assign structures to conformers because the differences in cross sections obtained for the three levels of theory were comparable to experimental error. Unlike the peptides in this work, Tureček’s peptides contain only one basic residue and the sites of protonation were the Arg side-chain and the N-terminus. Deprotonation of the phosphate was considered in conjunction with protonation of an amide group; however these structures collapsed with migration of the proton back to the phosphate.

In the current work, experimentally determined collision cross sections of doubly protonated ions of phosphopeptides APLpSFRGSLPKSYVK, APLSFRGSLPKpSYVK, APLpSFLGSLPKSYVK, and APLSFLGSLPKpSYVK were compared with model structures derived by molecular dynamics simulations with the aim of rationalizing observed ECD fragmentation. Different protonation patterns were considered in order to investigate the nature of any intramolecular interactions. The results suggest that for APLpSFRGSLPKSYVK, a salt-bridge structure is favored, whereas for APLSFRGSLPKpSYVK, ionic hydrogen bonds predominate.

## Experimental

### Materials

The peptides APLSFRGSLPKSYVK, APLpSFRGSLPKSYVK, APLSFRGSLPKpSYVK, APLSFLGSLPKSYVK, APLpSFLGSLPKSYVK, and APLSFLGSLPKpSYVK, where pS is phosphoserine, were synthesized by AltaBiosciences (Birmingham, UK) or GenicBio (Shanghai, China) and used without further purification.

### Electron Capture Dissociation Mass Spectrometry

The (phospho)peptide samples were prepared at a concentration of 2 pmol/μL in methanol:water:formic acid (49.5:49.5:1). All ECD mass spectrometry experiments were performed on a Thermo Fisher LTQ FT Ultra mass spectrometer (Bremen, Germany). Samples were injected by use of an Advion Biosciences Triversa Nanomate electrospray source (Ithaca, NY, USA) at a flow rate of ~200 nL/min. All mass spectra were acquired in the ICR cell with a resolution of 100,000 at *m*/*z* 400. Precursor ions were isolated in the linear ion trap and transferred to the ICR cell for ECD. AGC was 5 × 10^5^ with maximum fill time 1 s and the isolation width was *m*/*z* 3. Electrons for ECD were produced by an indirectly heated barium-tungsten cylindrical dispenser cathode (5.1 mm diameter, 154 mm from the cell, 1 mm off axis) (Heat-Wave Labs, Watsonville, CA, USA). Ions were irradiated with electrons for 420 ms at 5% energy. Each MS/MS ECD scan comprises four co-added microscans. ECD mass spectra shown comprise 30 averaged scans.

All data were analyzed using Xcalibur 2.1.0 software (Thermo Fisher Scientific), and manually searched for *a*, *b*, *c* ^*•*^/*c*, *y*, *z*/*z*′ fragment ions using ProteinProspector ver. 5.7.2 software (UCSF, San Francisco, CA, USA). Fragment ion relative abundances were calculated by dividing the abundance of each monoisotopic fragment ion by the sum of abundances of all fragment ions (including the charge-reduced species) within the mass spectrum and reported as %. Reported values are the mean of three repeats. *P* values were calculated to determine significance between fragment ion relative intensities using the Student’s *t*-test (*n* = 3).

### Traveling Wave Ion Mobility Spectrometry

The (phospho)peptide samples were dissolved in water/acetonitrile (1:1) containing 0.1% formic acid to a final concentration of 1 pmol/μL, and analyzed using a SYNAPT G2 HDMS mass spectrometer (Waters Corp., Milford, MA, USA) equipped with a nano-ESI source and a traveling-wave ion mobility cell (TW-IMS) maintained at 3 mbar of nitrogen. TW-IMS was operated at a wave velocity of 450 m/s and wave amplitude of 20, 25, and 30 V. CCS calibration was performed by the method described previously by Ruotolo et al.[30] Calibration standards included tryptic digest peptides from BSA, cytochrome *c*, and myoglobin. Literature values for all CCS calibrant peptides were obtained from the CCS reported by Clemmer et al. [31].

### Molecular Dynamics Simulations

Simulated annealing was performed by use of code developed in-house using AMBER 9 and the AMBER99SB force field [32]. Custom residues were made following RED III resp tool [33] and Gaussian 03 [34]. The phosphoserine force field was obtained from the Bryce Group, University of Manchester, UK (http://www.pharmacy.manchester.ac.uk/bryce/amber/). A total of three tiers of simulated annealing simulation were performed. In the first tier, alpha helical and extended backbone structures were created within AMBER 9 as the starting structures for the simulated annealing. A total of 1000 structures were simulated from each starting structure. In the second tier, 24 starting structures were randomly selected from the first tier, and each was subjected to simulated annealing, resulting in 1000 structures. For the third tier, again 24 structures were randomly selected from the second tier and simulated to create 1000 structures. A total of 50,000 structures were created. The MOBCAL trajectory method was used to calculate the CCS of the simulated structures [35, 36]. Simulated structures were filtered to within ±3% of the experimentally-derived CCS. The K-clust [37] clustering method was applied to the filtered structures.

## Results and Discussion

Previously, we have demonstrated the effect of phosphorylation on the electron capture dissociation behavior of peptides with the sequence APLSFRGSLPKSYVK [8]. A summary of the results is shown in Table 1. In each case, no ECD fragments were observed between the phosphorylated residue and the basic amino acids (either R6 or K11). We concluded that noncovalent interactions, either salt-bridges or ionic hydrogen bonds, were preventing the separation of any ECD fragments. To test this hypothesis, peptides were synthesized in which the arginine residue at position 6 was replaced with leucine and the ECD fragmentation recorded. The fragmentation observed is summarised in Table 1 and the relative fragment ion abundances are shown in Table 2, together with those obtained for the arginine-containing peptides. The ECD MS/MS spectra of the leucine-containing peptides are shown in Supplemental Figure 1. For clarity, throughout this paper the peptides are denoted R6pSX and L6pSX, where X is the residue number of the phosphoserine, for arginine- and leucine-containing peptides, respectively.

**Table 1:**
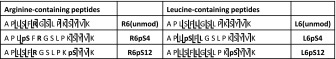
Summary of Fragments (*c* and *z* ions) Obtained Following ECD of (Phospho)peptides

Salient amino acid residues (pS, R, L) are shown in bold

Phosphorylation site, R6 and L6 is bolded for easy identification

**Table 2:** Relative Fragment Ion Abundance (Calculated as % of Total Fragment Ion Abundance) Following ECD of (Phospho)peptides (*n* = 3)

	R6pS4	R6S12	R6(unmod)	L6S4	L6S12	L6(unmod)
y_9_						0.70
y_10_					0.37	
z_7_^•^					0.59	5.14
z_7_					2.73	5.53
z_8_					0.78	1.83
z_9_					0.18	4.15
z_10_^•^		4.65	5.41		2.73	5.73
z_10_		5.82	4.44	0.92	3.62	4.30
z_11_^•^		3.92	4.36	1.25	1.24	3.71
z_11_		4.58	3.75	4.17	1.36	2.72
z_12_^•^-H_3_PO_4_					0.82	
z_12_ -H_3_PO_4_					0.42	
z_12_^•^	2.06	0.64	1.42	0.82	1.54	2.30
z_12_	1.39	0.26	1.16	0.66	1.15	1.60
z_13_^•^		0.54	1.42	0.26	0.98	
z_13_		0.92		0.35	0.65	
c_6_^•^			0.15			
c_6_			0.33			
c_7_^•^			0.51			
c_7_			0.75			
c_8_^•^			0.84			
c_8_			1.25			
c_10_^•^			0.43			
c_10_			1.35			
c_11_^•^	2.34		1.51	3.21	2.63	2.10
c_11_	3.63		2.25	3.90	2.29	6.34
c_12_^•^	3.16	3.24	1.31	2.53	4.72	
c_12_	7.39	6.51	6.38	5.34	6.87	4.69
c_13_	2.83	0.40	1.97	2.60	1.85	2.18
c_14_	5.27	4.34	2.96	5.48	5.11	5.11
[M + 2H^–^H_3_PO_4_]^+•^	3.61	1.68		3.09	3.97	
[M + H]^+^	1.61	1.12	1.39	2.23	2.49	1.04
[M + 2H]^+•^	16.86	13.16	11.47	15.33	11.39	8.69

The ECD behavior observed for the unmodified leucine-containing peptide, L6(unmod), reflects that observed previously for R6(unmod) in that fragmentation throughout the peptide sequence was observed. The distribution of *c* and *z* fragments differed between the two peptides, as would be expected: the ECD fragments of arginine-containing precursor ions typically contain the arginine residue [38]. In contrast, the ECD fragmentation of the phosphopeptides differs between the arginine- and leucine-containing peptides. Whereas R6pS4 showed no fragments between pS4 and K11, L6pS4 showed fragmentation in that region (z_10_, z_11_). It is interesting to note that the relative abundance of the z_11_ fragment of L6pS4 (C-terminal of the phosphoserine) is 3-fold greater than that from L6pS12 and ~2-fold that of L6(unmodified) (a Student’s *t*-test gives *P* = 0.001 and 0.002, respectively). L6pS12 showed fragmentation throughout the sequence, but ECD of R6pS12 resulted in no fragments between R6 and pS12. The relative abundance of the L6pS12 fragments were lower than those observed for L6(unmodified) with the exception of the fragments C-terminal of the phosphoserine c_12_/c_12_^•^ (*P* = 0.005). For all of the phosphopeptides, the relative abundance of the charge reduced species was greater than that observed for their unmodified counterparts (*P* < 0.01). The relative abundance of the peaks corresponding to [M + 2H – H_3_PO_4_]^+•^ were similar for L6pS4, L6PS12, and R6pS4. For R6pS12, the relative abundance of this fragment was approximately half that of the other phosphopeptides (*P* < 0.01 in all cases).

The results for L6pS12 appear to correlate with our hypothesis that noncovalent interactions are preventing separation of ECD fragments: If an interaction between R and pS12 limits the fragments detected for R6pS12, replacement of arginine with leucine (which does not have the capacity for electrostatic interaction with phosphoserine) should result in the appearance of additional fragments. The results for L6pS4, however, seem at odds with our hypothesis. If the noncovalent interaction responsible for the observed ECD in R6pS4 is between pS4 and K11, why is it not retained in L6pS4, and why do we not observe similar ECD behavior?

In order to address the question posed above, and to probe the nature of the noncovalent interactions (salt-bridges, ionic hydrogen bonds) in these phosphopeptides, we combined molecular dynamics simulations with traveling wave ion mobility spectrometry measurements to gain insight into the structures of the phosphopeptides.

**Figure 1:**
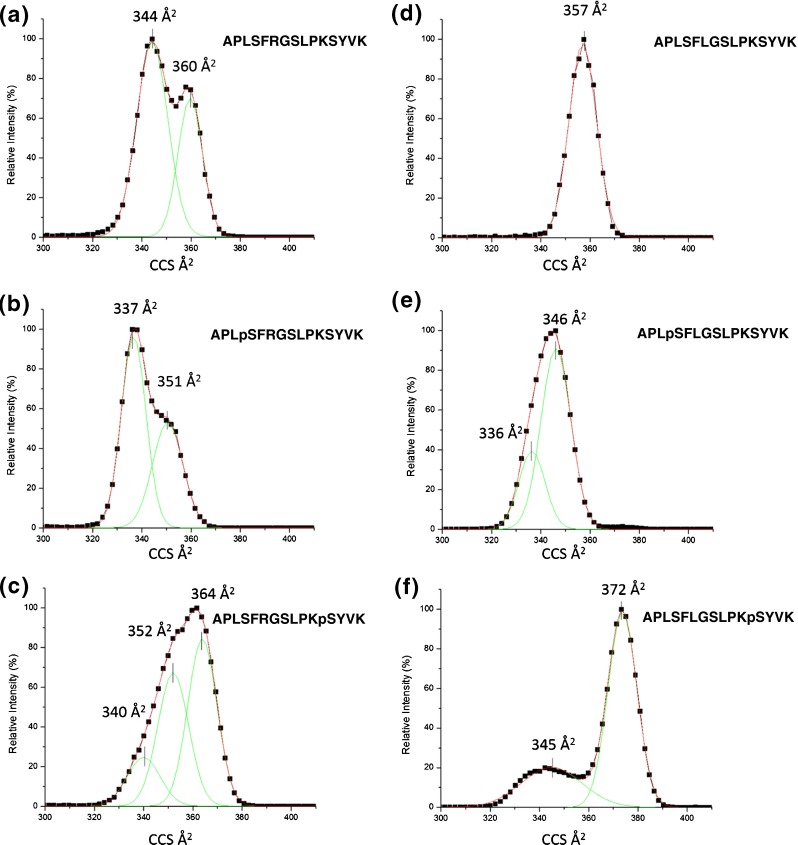
Mobility profiles for [M + 2H]^2+^ ions of R6(unmod) (**a**), R6pS4 (**b**), R6pS12 (**c**), L6(unmod) (**d**), L6pS4 (**e**), and L6pS12 (**f**)

Figure 1 shows the mobility profiles for [M + 2H]^2+^ ions of R6(unmod), R6pS4, R6pS12, L6(unmod), L6pS4, and L6pS12. The ion-neutral CCS of the phosphopeptides and their comparison with the random coil trend line and the unmodified peptides are shown in Table 3. The deviations for both L6pSX and R6pSX are positive with respect to the random coil trend line, which suggests that these ions have extended conformations [39]. It is important, however, to compare the CCS of the phosphopeptides with that of the unmodified counterpart. In that respect, both the R6 peptides show negative deviations consistent with compaction as a result of phosphorylation. L6pS4 also shows a negative deviation compared with L6(unmod), again suggesting compaction as a result of phosphorylation; however, L6pS12 shows a positive deviation, in agreement with the ECD experiments.

**Table 3:** The Ion-Neutral CCS for L6pSX and R6pSX Phosphopeptides and Their Non-Phosphorylated Analogues and CCS Comparison with the Random Coil Peptide Trendline and Unmodified Peptides

Peptide	Sequence			
		CCS (Å^2^)^a^	ΔCCS to RC(%)^b^	ΔCCS to unmod(%)^c^
L6pS4	APL**pS**F**L**GSLPKSYVK	346	+3.0	–6.4
L6pS12	APLSF**L**GSLPK**pS**YVK	372	+10.8	+1.4
L6(unmod)	APLSF**L**GSLPKYVK	357	+9.4	—
R6pS4	APL**pS**F**R**GSLPKSYVK	351	+2.7	–7.6
R6pS12	APLSF**R**GSLPK**pS**YVK	364	+6.5	–3.8
R6(unmod)	APLSF**R**GSLPKSYVK	360	+10.3	—

^a^ The CCS for the least compact conformer peak

^b^ The percentage deviation of CCS from that for a random coil peptide. The reference points for all CCS values were calculated based on the regression line and the molecular weight of each phosphopeptide

^c^ The CCS deviation relative to the unmodified peptide

As described above, molecular dynamics simulations were performed for each of the phosphopeptides. Candidate structures were generated by simulated annealing and their CCS calculated via the trajectory method in MOBCAL. The structures were subsequently filtered to with ±3% of the experimentally derived CCS, and the filtered structures subjected to *k*-means cluster analysis. To address the question of the nature of any intramolecular noncovalent interactions, for each set of peptides two protonation patterns were considered. For the R6 peptides: (a) protonation of R6, K11, and K15 together with deprotonation of the phospho-group for an overall charge of +2 (for which we might anticipate salt-bridge formation), denoted herein as (R+, K11+, K15+, PO_x_–); and (b) protonation of R6 and K15 with neutral phosphate (ionic hydrogen bonds anticipated), denoted herein as (R+, K15+, PO_x_0). For the L6 peptides: (a) protonation of the N-terminus, K11 and K15, and deprotonation of the phospho-group (NT+, K11+, K15+, PO_x_–), and (b) protonation of K11 and K15 with neutral phosphate (K11+, K15+, PO_x_0). (Note that protonation of the N-terminus was not considered when modeling the R6 peptides: N-acetylated versions of the phosphopeptides showed identical ECD fragmentation behavior to that of the non-acetylated R6 peptides (data not shown), suggesting that protonation of the N-terminus is not involved).

**Figure 2:**
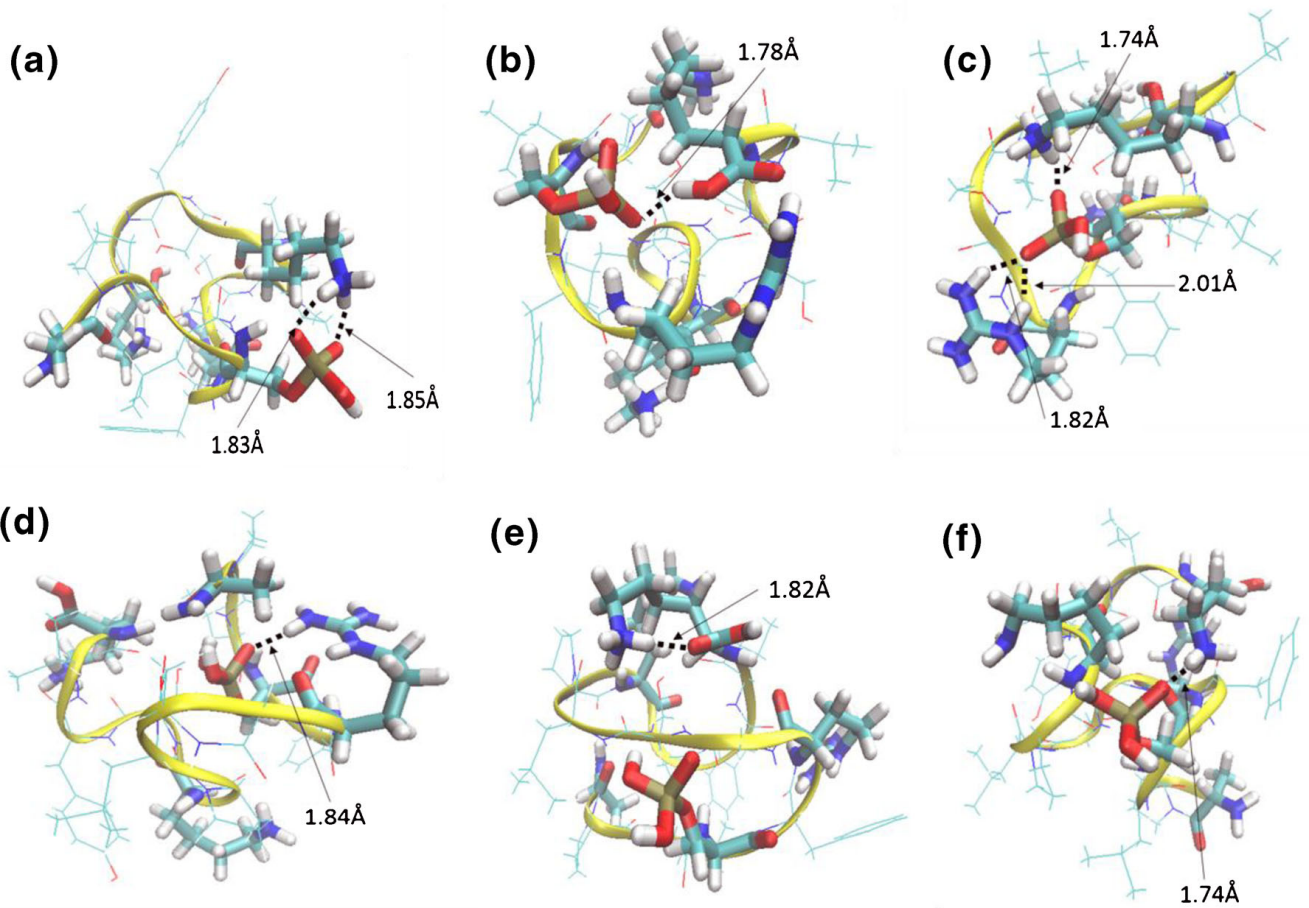
Model structures for the 337 Å^2^ conformer of R6pS4 (**APLpSFRGSLPKSYVK**) representative of the three most populated clusters (**a**, **b**, **c**) for (R+, K11+, K15+, POx–) and (**d**, **e**, **f**) (R+, K15+, POx0)

The mobility data for R6pS4 (Figure 1b) suggests that there are two conformers, one of CCS 337 Å^2^ and one of 351 Å^2^. The modeled structures closest to the centroid of the three most populated clusters for conformer with CCS 337 Å^2^ of R6pS4 (R+, K11+, K15+, POx–) are shown in Figure 2a, b, and c. Figure 2a comprises ~16% of the filtered structures (total structures 3730); Figure 2b comprises ~13%, and Figure 2c comprises ~10%. The structures were analyzed according to the proximity between the basic amino acid side chains and the phosphate group. Ion pairs may be considered salt-bridges if the distance between charged atoms is less than 4 Å [15]. The results of the distance analyses are summarized in Table 4. For cluster 1 (Figure 2a), the distance between pS4 and K11 was <4 Å, suggesting the presence of a salt-bridge between these residues. This observation correlates well with the ECD data, assuming that the salt-bridge survives the electron capture/dissociation event. The distances between pS4-R6 and pS4K15 were both >4 Å. For cluster 2 (Figure 2b), all of the protonated residues were >4 Å distance from pS4. For cluster 3 (Figure 2c), pS4-R6 and pS4-K15 were <4 Å, suggesting the presence of salt-bridges. Again, the presence of these salt-bridges would limit the observed ECD fragmentation.

**Table 4:** Distance Analysis of Model Structures of R6pSX and L6pSX peptides. ‘Y’ Indicates Distance of <4 Å from Phosphoserine

	R6	K11	K15		K11	K15
**R6pS4**(R+, K11+, K15+, PO_x_–)				**L6pS4** (N+, K11+, K15+, PO_x_–)		
337 Å^2^ Structure 1	-	Y	-	336 Å^2^ Structure 1	Y	Y
337 Å^2^ Structure 2	-	-	-	336 Å^2^ Structure 2	-	Y
337 Å^2^ Structure 3	Y	-	Y	336 Å^2^ Structure 3	-	-
**R6pS4** (R+, K15+, PO_x_0)				**L6pS4** (K11+, K15+, PO_x_0)		
337 Å^2^ Structure 1	Y	-	-	336 Å^2^ Structure 1	-	-
337 Å^2^ Structure 2	-	-	-	336 Å^2^ Structure 2	Y	-
337 Å^2^ Structure 3	Y	-	-	336 Å^2^ Structure 3	Y	-
**R6pS4**(R+, K11+, K15+, PO_x_–)				**L6pS4**(N+, K11+, K15+, PO_x_–)		
351 Å^2^ Structure 1	Y	-	-	346 Å^2^ Structure 1	Y	-
351 Å^2^ Structure 2	-	Y	Y	346 Å^2^ Structure 2	-	Y
351 Å^2^ Structure 3	-	-	Y	346 Å^2^ Structure 3	-	Y
**R6pS4** (R+, K15+, PO_x_0)				**L6pS4** (K11+, K15+, PO_x_0)		
351 Å^2^ Structure 1	-	Y	-	346 Å^2^ Structure 1	-	-
351 Å^2^ Structure 2	Y	-	-	346 Å^2^ Structure 2	Y	-
351 Å^2^ Structure 3	-	-	-	346 Å^2^ Structure 3	-	-
**R6pS12** (R+, K11+, K15+, PO_x_–)				**L6pS12** (N+, K11+, K15+, PO_x_–)		
340 Å^2^ Structure 1	Y	Y	-	372 Å^2^ Structure 1	Y	-
340 Å^2^ Structure 2	-	Y	Y	372 Å^2^ Structure 2	-	-
340 Å^2^ Structure 3	Y	Y	-	372 Å^2^ Structure 3	Y	-
**R6pS12** (R+, K15+, PO_x_0)				**L6pS12** (K11+, K15+, POx0)		
340 Å^2^ Structure 1	-	-	-	372 Å^2^ Structure 1	-	-
340 Å^2^ Structure 2	-	Y	-	372 Å^2^ Structure 2	Y	-
340 Å^2^ Structure 3	-	-	Y	372 Å^2^ Structure 3	-	-
**R6pS12** (R+, K11+, K15+, PO_x_–)						
352 Å^2^ Structure 1	-	Y	Y			
352 Å^2^ Structure 2	-	Y	Y			
352 Å^2^ Structure 3	-	Y	Y			
**R6pS12** (R+, K15+, PO_x_0)						
352 Å^2^ Structure 1	-	-	Y			
352 Å^2^ Structure 2	Y	-	-			
352 Å^2^ Structure 3	-	-	Y			
**R6pS12** (R+, K11+, K15+, PO_x_–)						
364 Å^2^ Structure 1	-	-	Y			
364 Å^2^ Structure 2	-	Y	-			
364 Å^2^ Structure 3	-	Y	Y			
**R6pS12** (R+, K15+, POx0)						
364 Å^2^ Structure 1	Y	-	Y			
364 Å^2^ Structure 2	Y	-	-			
364 Å^2^ Structure 3	Y	-	-			

The structures for conformer with CCS 337 Å^2^ of R6pS4 (R+, K15 + PO_x_0) are shown in Figure 2d, e, and f. The cluster populations are ~17%, ~16%, and ~7% of the 6351 total filtered structures, respectively. The structures were again analyzed according to the proximity between the basic amino acid chains and the phosphate group. For these structures, we are interrogating the presence (or otherwise) of ionic hydrogen bonds rather than salt-bridges; however, the limit of 4 Å was retained for consistency. For cluster 1 (Figure 2d) and cluster 3 (Figure 2f), the distance between pS4 and R6 was <4 Å, suggesting the presence of an ionic hydrogen bond. In both cases, the distances between pS4 and the lysine residues was >4 Å. Cluster 2 (Figure 2e) had no interaction distances <4 Å. Based on these models, the ECD fragmentation would be predicted to be extensive even if the pS4–R6 ionic hydrogen bond were maintained throughout the ECD event. For this conformer, therefore, the salt-bridge model is the better descriptor of experimental findings.

Modeled structures for the second conformer of R6pS4 (351 Å^2^) are shown in Supplemental Figure 2. The structures for (R6+, K11+, K15+, PO_x_–) are shown in Supplemental Figure 2A, B, and C, with populations ~21%, ~8%, and ~7% of the total filtered structures (16,758) respectively. Distance analysis revealed that for cluster 1 (Supplemental Figure 2A) a salt-bridge exists between pS4 and R6 only, and that K11 was buried in the peptide backbone. Based on this, extensive ECD fragmentation would be predicted contrary to what is observed experimentally. The remaining structures show salt-bridges between pS4-K11 and pS4-K15 (Supplemental Figure 2B) or pS4-K15 (Supplemental Figure 2C), both of which would lead to reduced ECD fragmentation. The structures for (R6+, K15+, PO_x_0) are shown in Supplemental Figure 2D, E, and F, with populations ~10%, ~7%, and ~6%, respectively. Distance analysis reveals ionic hydrogen bonds between pS4 and R6 in structure 2 (Supplemental Figure 2E) only. The distance between pS4 and K11 in structure 1 is <4 Å; however, this interaction would be a (non-ionic) hydrogen bond and would be unlikely to survive ECD. The modeled structures for (R6+, K15+, PO_x_0) would lead one to predict extensive ECD, however, that is not observed experimentally. Overall, despite the outlier that is (R6+, K11+, K15+, PO_x_–) cluster 1, the salt-bridge structures again appear better descriptors of experimental behavior.

It is interesting to note that for each of the structures for R6pSX in which the phosphate is negative, interactions between the lysine side chain and phosphate residues are favored, whereas when the phosphate is neutral, interactions between the arginine side chain and the phosphate predominate. Arginine is known to confer greater stability to proteins than lysine as a result of the guanidinium group’s ability to interact in three directions compared with the single direction available to the lysine’s ammonium group [16, 40], allowing arginine to participate in a greater number of electrostatic interactions. It has been suggested that arginine electrostatic interactions in the condensed phase are more stable than those of lysine because of the higher pKa of arginine [40]. Conversely, Jungwirth has shown that unlike the lysine ammonium group, the diffuse guanidinium group will pair with other guanidinium groups in water [41]. Meot-Ner [42] has shown that ionic hydrogen bond strength depends on the relative proton affinity of the interacting partners. A donor with higher relative proton affinity will transfer a proton less efficiently to the acceptor. The intrinsic PA of arginine is reported to be between 242.8 and 245.2 kcal/mol and that of lysine is between 225.5 and 230.3 kcal/mol [43], although the actual PA will depend on peptide ion structure .

The mobility data for R6pS12 (Figure 1c) suggests three conformers of CCS 340 Å^2^, 352 Å^2^, and 364 Å^2^. The modeled structures for the three conformers are shown in Supplemental Figures 3, 4, and 5. Consider the structures for conformer of CCS 340 Å^2^ (R6+, K11+, K15+, PO_x_–) (Supplemental Figure 3 A, B, C). The populations of the clusters are ~15%, ~10%, and ~8% of the 4943 total filtered structures, respectively. Distance analysis reveals salt bridges between R6-pS12 and K11-pS12 in the structures shown in Supplemental Figure 3A and C, which correlates very well with the observed ECD patterns. The structure shown in Supplemental Figure 3B has salt-bridges between K11-pS12 and pS12-K15, for which we might expect some fragmentation central to the peptide sequence but which is not observed. For each of these structures, the phosphate group is participating in multiple electrostatic interactions. Similar behavior (i.e., multidentate binding), was observed by Breuker and co-workers in their work on structural evolution of cytochrome *c* ions during electrospray [44]. Distance analysis of the structures for the same conformer in protonation state (R6+, K15+, PO_x_0) (see Supplemental Figure 3 D, E, F; populations ~13%, ~8%, and ~8% of the 9308 total filtered structures) suggest (non-ionic) hydrogen bonds between K11 and pS12 in the structure shown in Supplemental Figure 3E, and an ionic hydrogen bond between pS12 and K15 in the structure shown in Supplemental Figure 3F, none of which correlate with the observed ECD fragmentation. For this conformer, therefore, the salt-bridge structures are the best models to explain the ECD behavior. Nevertheless, this conformer is the minor of the three and it is likely that conformers with CCS 352 Å^2^ and 364 Å^2^ will make a bigger contribution to observed ECD patterns.

For the conformer with CCS 352 Å^2^ (Supplemental Figure 4), distance analysis of the structures in protonation state (R6+, K11+, K15+, PO_x_–) reveals salt bridges between K11-pS12 and pS12-K15 in each case. (Cluster populations are ~11%, ~8%, and ~8% of 15,833 total filtered structures). None showed salt bridges between R6 and pS12. In each structure, the arginine protrudes away from the phosphate group, similar to the observations of Tureček and co-workers [28]. Clearly, these models cannot explain observed ECD behavior. Two out of three structures in protonation state (R6+, K15+, PO_x_0) are also at odds with experimental observations. Those structures, shown in Supplemental Figure 4D and F, show ionic hydrogen bonds between pS12 and K15. (Cluster populations are ~15%, ~13%, and ~8% of 3595 total structures). For this conformer, neither set of model structures accurately describes the ECD. It is worth noting that in this work we have only considered two protonation states, and there may be third (or more), which account for this conformer. Nevertheless, as mentioned above, the ECD of doubly protonated ions of N-acetylated R6pS12 suggests that protonation of the N-terminus does not play a significant role.

Distance analysis of the structures for the third conformer (CCS 364 Å^2^) (Supplemental Figure 5) in protonation state (R6+, K11+, K15+, PO_x_–; total population of 24,583) reveals salt-bridges between pS12 and K15 (Supplemental Figure 5A), population ~10%), K11 and pS12 (Supplemental Figure 5B, population ~7%) or both pS12 and K15; K11 and pS12 (Supplemental 5C, population ~5%). Again in each case, the arginine residue protrudes away from the phosphate group. In contrast, all of the structures (cluster populations ~11%, ~6%, ~5% of total 1249 filtered structures) in protonation state (R6+, K15+, PO_x_0) show ionic hydrogen bonds between R6 and pS12 (with an additional ionic hydrogen bond between pS12 and K15 in the structure shown in Supplemental Figure 5D). That is, for this conformer, the structures with neutral phosphate and ionic hydrogen bonds are the most appropriate models for observed ECD fragmentation.

The mobility data for L6pS4 (Figure 1e) suggests the presence of two conformers of CCS 336 Å^2^ and 346 Å^2^. The ECD fragmentation observed for this peptide is more extensive than for R6pS4 but less extensive than for L6(unmod), suggesting that some intramolecular interactions involving the phosphate group may be present but are insufficiently strong to completely prevent separation of fragments. The mobility data suggest compaction of structure on phosphorylation. Model structures were generated as described above. The structures for the conformer with CCS 336 Å^2^ and protonation state (N+, K11+, K15+, PO_x_–) are shown in Supplemental Figure 6 A, B, and C. Cluster populations are ~27%, ~15%, and ~14% of the total population of 521 filtered structures, respectively. Salt bridges are present in the structures between pS4 and K11 and between pS4 and K15 (Supplemental Figure 6A), and between pS4 and K15 (Supplemental Figure 6B). That is, over 40% of the structures display salt-bridges. The third structure has no salt bridges. In the (K11+, K15+, PO_x_0) protonation state (Supplemental Figure 6 D, E, and F) (total population 6437 filtered structures), the structures shown in Supplemental Figure 6E and F (~8% and ~8%) show ionic hydrogen bonds between pS4 and K11, and the structure shown in Supplemental Figure 6D (~13%) shows no interactions between the phosphate and lysine side-chains of <4 Å.

Structures for the more abundant conformer (CCS 346 Å^2^) are shown in Supplemental Figure 7. In protonation state (N+, K11+, K15+, PO_x_–) (total population of 12,280 filtered structures), salt bridges exist between pS4 and K11 (Supplemental Figure 7A, ~9%); between pS4 and K15 (Supplemental Figure 7B and C, ~8% and ~6%, respectively). In protonation state (K11+, K15+, PO_x_0) (total population 17,342 structures), there are no interactions between the phosphate and K11 or K15 in the structures shown in Supplemental Figure 7D, (~31%) and Supplemental Figure 7F (~8%) and an ionic hydrogen bond present between pS4 and K11 in the structure shown in Supplemental Figure 7E (~22%).

The mobility data for L6pS12 (Figure 1f) suggests one major conformer of CCS 372 Å^2^ and one minor of CCS 345 Å^2^. The major conformer, which is assumed to make the major contribution to ECD, shows no compaction on phosphorylation, suggesting no structurally significant intramolecular interactions involving the phosphate group, correlating well with the ECD data. The modeled structures for the conformer with CCS 372 Å^2^ in both protonation states are shown in Supplemental Figure 8. These structures did not form highly populated clusters [~8%, ~7%, and ~5% for (N+, K11+, K15+, PO_x_–) (total population 11,759 structures) and ~5%, ~4%, and ~4% for (K11+, K15+, POx0) (total population 18,230 structures)]. For protonation state (N+, K11+, K15+, PO_x_–), salt-bridges were present between K11 and pS12 (Supplemental 8A); and between K11 and pS12; and pS12 and K11 (Supplemental Figure 8C). (The interaction indicated in Supplemental Figure 8B is between pS12 and the N-terminus). For protonation state (K11+, K15+, PO_x_0) ionic hydrogen bonds were present between K11 and pS12 (Supplemental Figure 8E). Any of these model structures could explain the observed ECD fragmentation.

In summary, for R6pS4, the model structures in which salt-bridges exist between pS4 (deprotonated) and K11 best fit the ECD data. For R6pS12, the model structures in which ionic hydrogen bonds exist between R6 and pS12 are the best fit. The question arises: Why do we see a difference in propensity to form salt-bridges in these isomeric phosphopeptides? Williams et al. [20] have shown that the presence of multiple (adjacent) arginine residues stabilizes the salt-bridge structures in dipeptide cations. It is possible, therefore, that the presence of arginine at *i + 2* from pS stabilizes the salt-bridge between pS4 and K11 in R6pS4. Salt-bridge (pS-K) stabilization by arginine would also explain the results for L6pS4: the more extensive fragmentation observed for L6pS4 suggests that any intramolecular interactions are less likely to survive electron capture (i.e., that no salt-bridges exist). Nevertheless, ion mobility data does suggest compaction on phosphorylation of the leucine-containing peptide, which may simply reflect the strength of pS–K interactions in comparison to pS–R interactions.

## Conclusion

We have shown that traveling wave ion mobility spectrometry and molecular dynamics simulations provide insight into the ECD behavior of a suite of phosphopeptides. The results suggest that for doubly protonated ions of APLpSFRGSLPKSYVK, a salt-bridge structure is favored in which the phosphate is deprotonated and forms an electrostatic interaction with the lysine at position 11. The salt-bridge appears to be stabilized by the presence of arginine at *i + 2*, although the arginine does not necessarily participate in the interaction. This conclusion is supported by the more extensive ECD fragmentation and ion mobility data observed for the analogous leucine-containing peptide. For doubly protonated ions of APLSFRGSLPKpSYVK, the best descriptor of ECD behavior arises when the phosphate is neutral and electrostatic interactions (ionic hydrogen bonds) exist between the arginine side-chain and phosphate group.

## Electronic supplementary material

Below is the link to the electronic supplementary material.

ESM 1 (DOCX 13 kb)

ESM 2 (PPTX 115 kb)

ESM 3 (PPTX 116 kb)

ESM 4 (PPTX 793 kb)

ESM 5 (PPTX 752 kb)

ESM 6 (PPTX 749 kb)

ESM 7 (PPTX 759 kb)

ESM 8 (PPTX 770 kb)

ESM 9 (PPTX 739 kb)

ESM 10 (PPTX 708 kb)
