# Supplementary material for: Probing the Electron Capture Dissociation Mass Spectrometry of Phosphopeptides with Traveling Wave Ion Mobility Spectrometry and Molecular Dynamics Simulations
Source: J Am Soc Mass Spectrom. 2015 Apr 2;26(6):1004–13. doi: 10.1007/s13361-015-1094-1 (PMC4422852; doi:10.1007/s13361-015-1094-1)
Supplement: Supplementary file 1 — (DOCX 13 kb) [file 13361_2015_1094_MOESM1_ESM.docx]

**Probing the electron capture dissociation mass spectrometry of phosphopeptides with travelling wave ion mobility spectrometry and molecular dynamics simulations**

Doyong Kim^1^, Pei-Jing Pai^1^, Andrew W. Jones^2^, Andrew J. Creese^2^,

David H. Russell^1^* and Helen J. Cooper^2^*

**Supplemental information**

**Supplemental Figures:**

**Supplemental figure 1:** ECD MS/MS spectra of leucine-containing peptides (A) L6(unmodified); (B) L6pS4 and (C) L6pS12

**Supplemental figure 2:** Model structures for the 351 Å^2^  conformer of R6pS4 representative of the three most populated clusters (**A, B, C**) for (R+,K11+,K15+,POx-) and (**D, E, F**) (R+,K15+,POx0).

**Supplemental figure 3:** Model structures for the 340 Å^2^  conformer of R6pS12 representative of the three most populated clusters (**A, B, C**) for (R+,K11+,K15+,POx-) and (**D, E, F**) (R+,K15+,POx0).

**Supplemental figure 4:** Model structures for the 352 Å^2^  conformer of R6pS12 representative of the three most populated clusters (**A, B, C**) for (R+,K11+,K15+,POx-) and (**D, E, F**) (R+,K15+,POx0).

**Supplemental figure 5:** Model structures for the 364 Å^2^  conformer of R6pS12 representative of the three most populated clusters (**A, B, C**) for (R+,K11+,K15+,POx-) and (**D, E, F**) (R+,K15+,POx0).

**Supplemental figure 6:** Model structures for the 336 Å^2^  conformer of L6pS4 representative of the three most populated clusters (**A, B, C**) for (N+,K11+,K15+,POx-) and (**D, E, F**) (K11+,K15+,POx0).

**Supplemental figure 7:** Model structures for the 346 Å^2^  conformer of L6pS4 representative of the three most populated clusters (**A, B, C**) for (N+,K11+,K15+,POx-) and (**D, E, F**) (K11+,K15+,POx0).

**Supplemental figure 8:** Model structures for the 372 Å^2^  conformer of L6pS12 representative of the three most populated clusters (**A, B, C**) for (N+,K11+,K15+,POx-) and (**D, E, F**) (K11+,K15+,POx0).
