# Supplementary material for: Probing the Electron Capture Dissociation Mass Spectrometry of Phosphopeptides with Traveling Wave Ion Mobility Spectrometry and Molecular Dynamics Simulations
Source: J Am Soc Mass Spectrom. 2015 Apr 2;26(6):1004–13. doi: 10.1007/s13361-015-1094-1 (PMC4422852; doi:10.1007/s13361-015-1094-1)
Supplement: Supplementary file 2 — (PPTX 115 kb) [file 13361_2015_1094_MOESM2_ESM.pptx]

## Slide 1
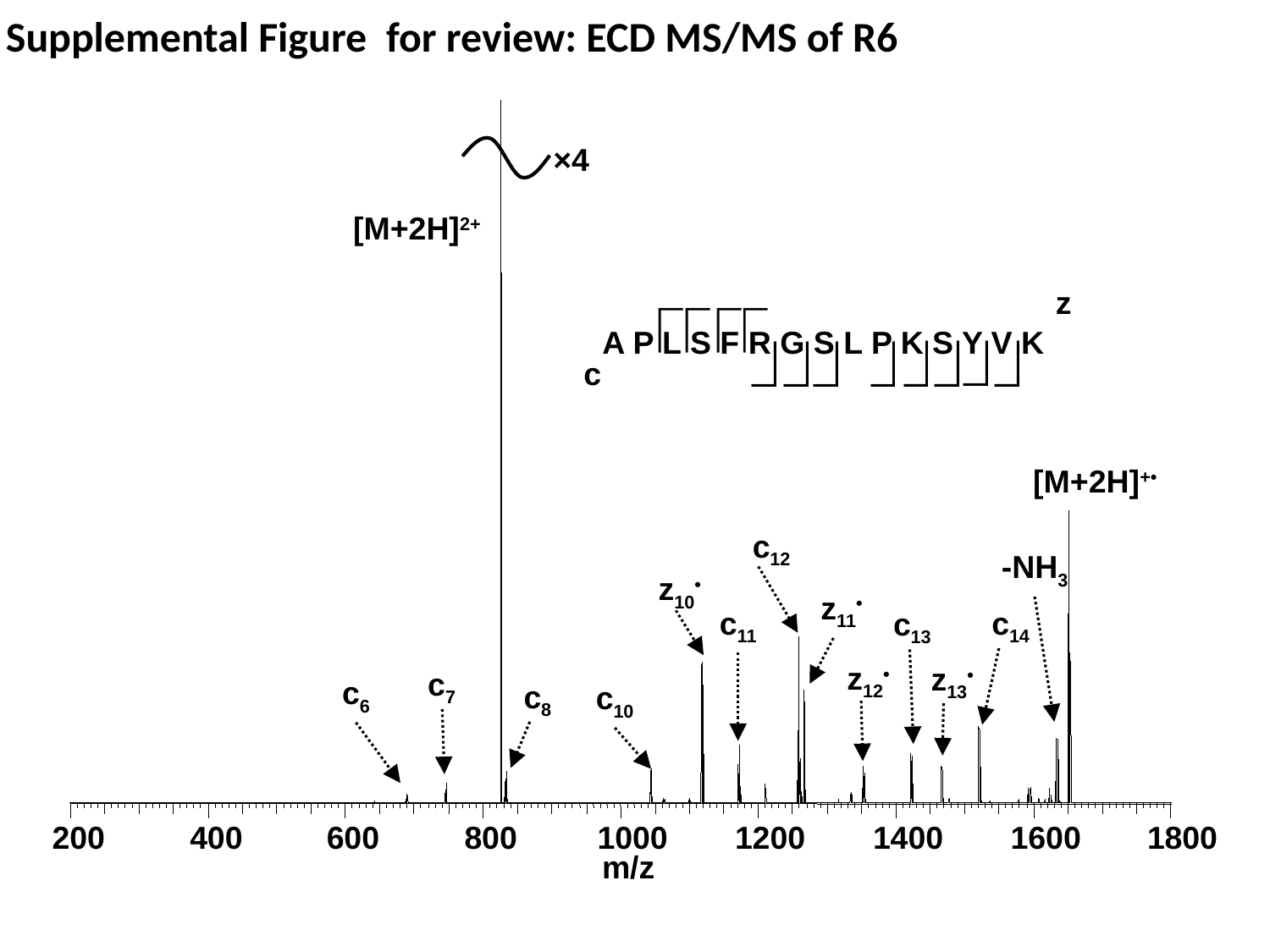

Supplemental Figure for review: ECD MS/MS of R6
200
400
600
800
1000
1200
1400
1600
1800
m/z
×4
[M+2H]2+
z
A P L S F R G S L P K S Y V K
c
[M+2H]+•
c12
-NH3
z10•
z11•
c11
c14
c13
z12•
z13•
c7
c6
c8
c10

## Slide 2
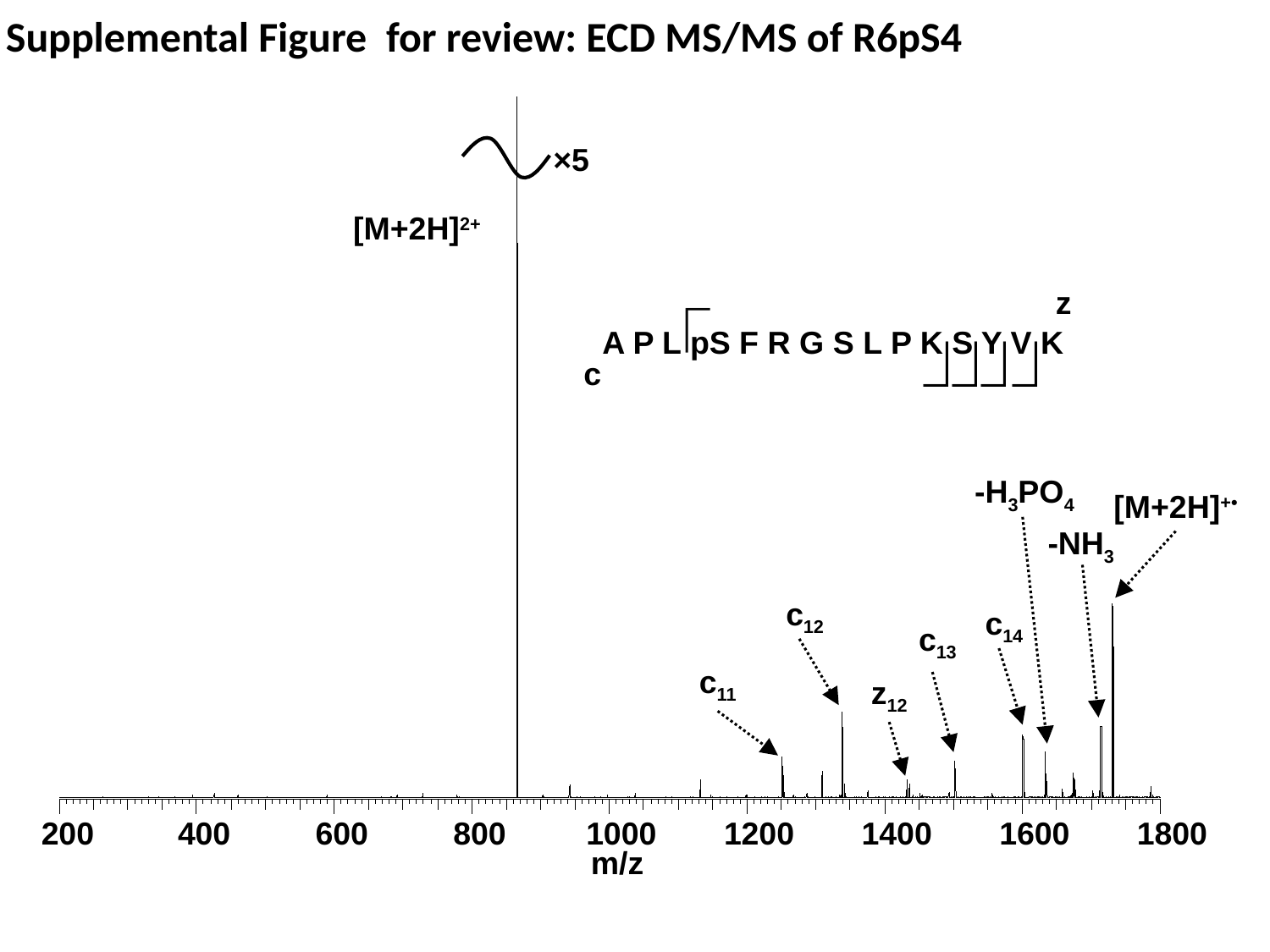

Supplemental Figure for review: ECD MS/MS of R6pS4
200
400
600
800
1000
1200
1400
1600
1800
m/z
×5
[M+2H]2+
z
A P L pS F R G S L P K S Y V K
c
-H3PO4
[M+2H]+•
-NH3
c12
c14
c13
c11
z12

## Slide 3
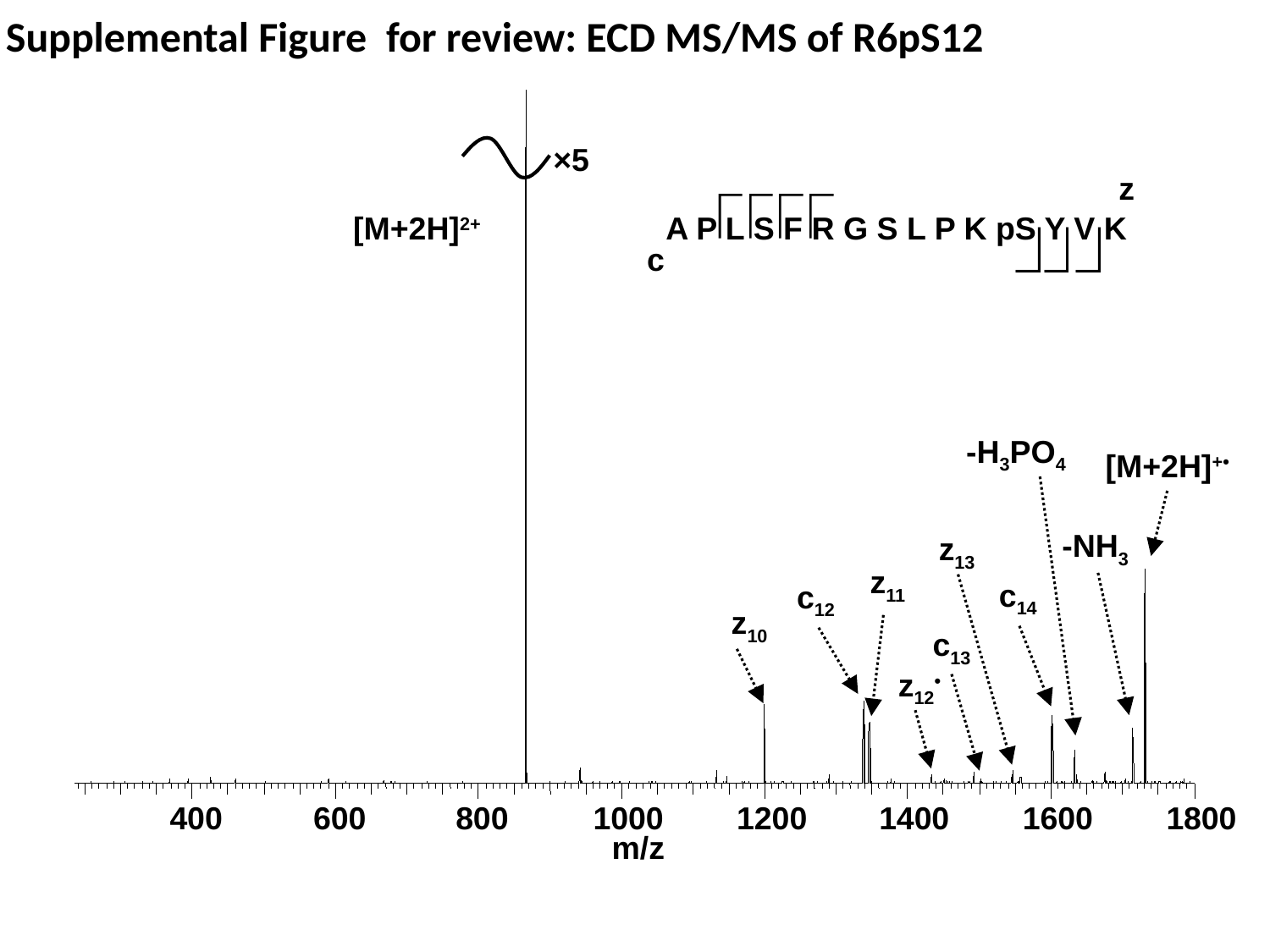

Supplemental Figure for review: ECD MS/MS of R6pS12
400
600
800
1000
1200
1400
1600
1800
m/z
×5
z
[M+2H]2+
A P L S F R G S L P K pS Y V K
c
-H3PO4
[M+2H]+•
-NH3
z13
z11
c14
c12
z10
c13
z12•
