# Supplementary material for: Probing the Electron Capture Dissociation Mass Spectrometry of Phosphopeptides with Traveling Wave Ion Mobility Spectrometry and Molecular Dynamics Simulations
Source: J Am Soc Mass Spectrom. 2015 Apr 2;26(6):1004–13. doi: 10.1007/s13361-015-1094-1 (PMC4422852; doi:10.1007/s13361-015-1094-1)
Supplement: Supplementary file 3 — (PPTX 116 kb) [file 13361_2015_1094_MOESM3_ESM.pptx]

## Slide 1
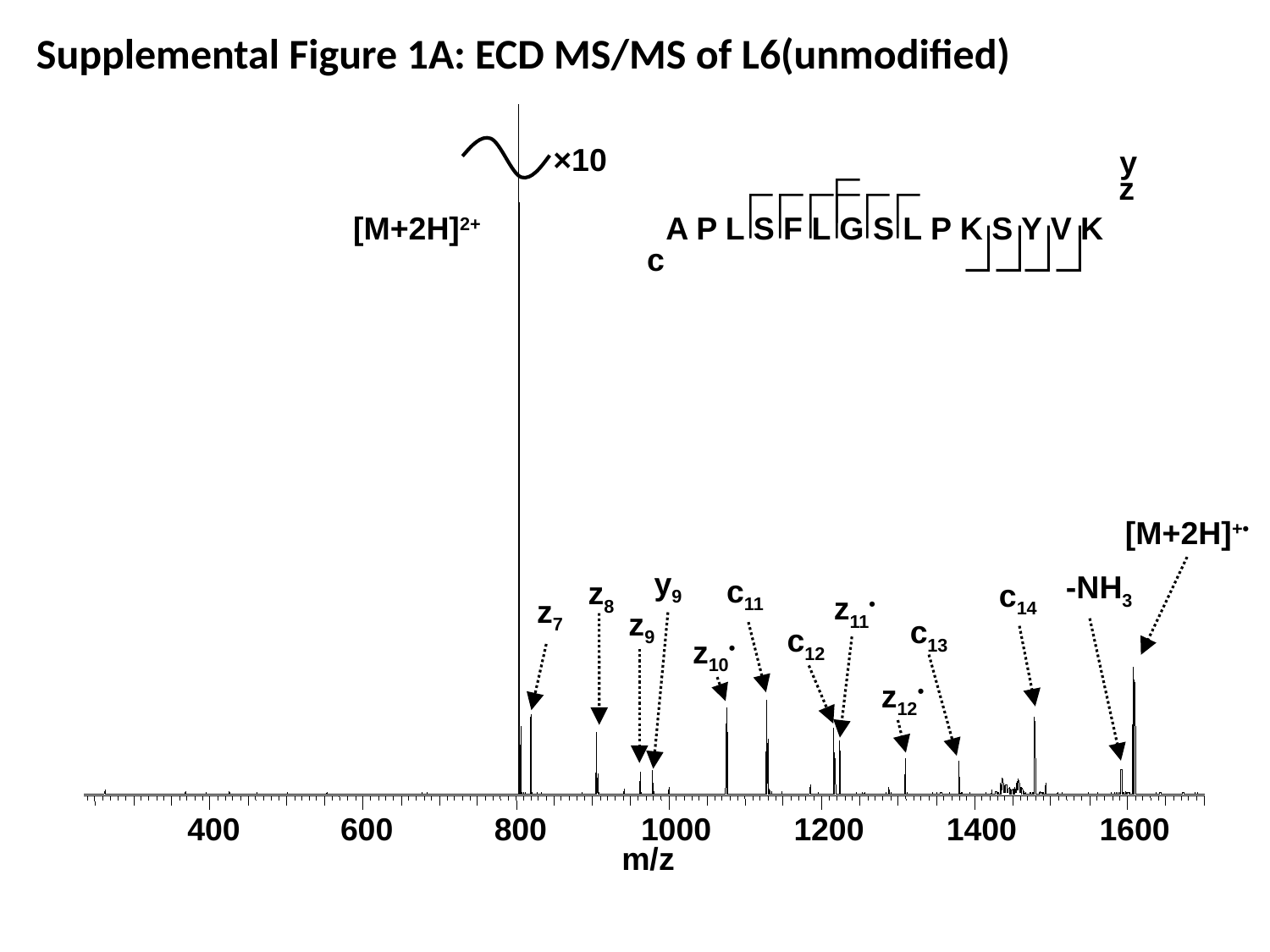

Supplemental Figure 1A: ECD MS/MS of L6(unmodified)
400
600
800
1000
1200
1400
1600
m/z
×10
y
z
[M+2H]2+
A P L S F L G S L P K S Y V K
c
[M+2H]+•
y9
-NH3
c11
z8
c14
z11•
z7
z9
c13
c12
z10•
z12•

## Slide 2
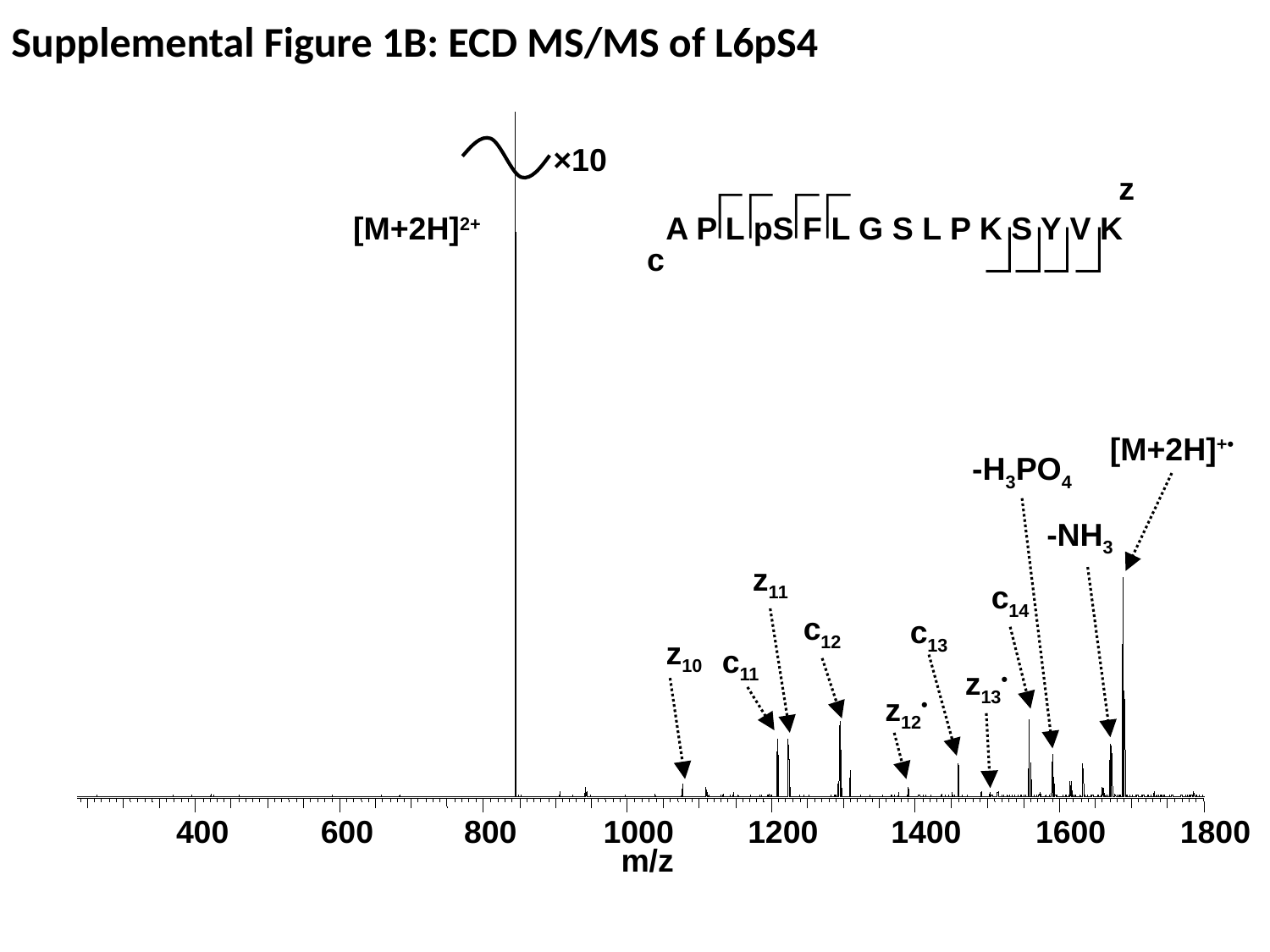

Supplemental Figure 1B: ECD MS/MS of L6pS4
400
600
800
1000
1200
1400
1600
1800
m/z
×10
z
[M+2H]2+
A P L pS F L G S L P K S Y V K
c
[M+2H]+•
-H3PO4
-NH3
z11
c14
c12
c13
z10
c11
z13•
z12•

## Slide 3
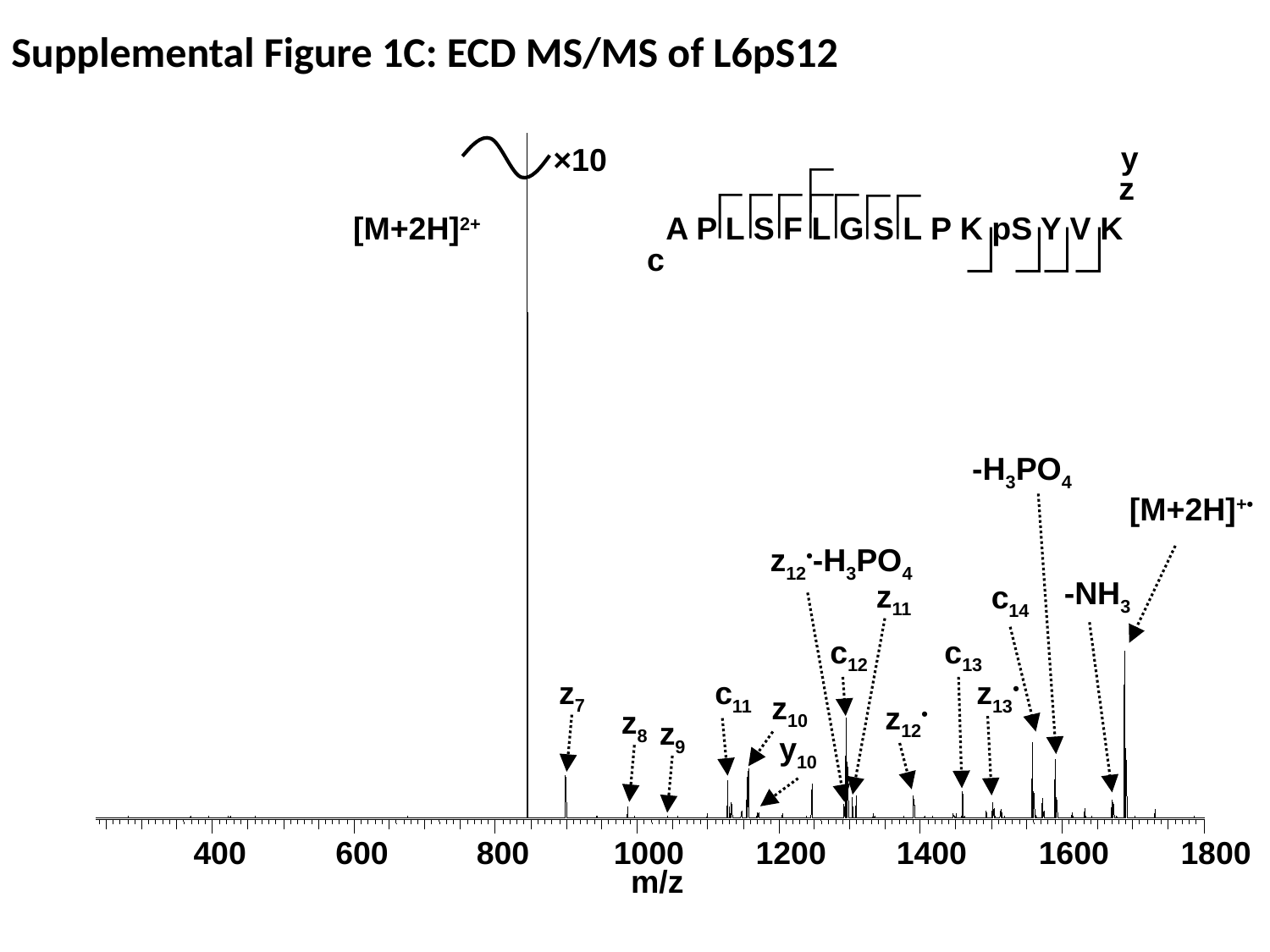

Supplemental Figure 1C: ECD MS/MS of L6pS12
y
400
600
800
1000
1200
1400
1600
1800
m/z
×10
z
[M+2H]2+
A P L S F L G S L P K pS Y V K
c
-H3PO4
[M+2H]+•
z12•-H3PO4
-NH3
z11
c14
c12
c13
z7
c11
z13•
z10
z12•
z8
z9
y10
