# Supplementary figures and images for: Probing the Electron Capture Dissociation Mass Spectrometry of Phosphopeptides with Traveling Wave Ion Mobility Spectrometry and Molecular Dynamics Simulations
Source: J Am Soc Mass Spectrom. 2015 Apr 2;26(6):1004–13. doi: 10.1007/s13361-015-1094-1 (PMC4422852; doi:10.1007/s13361-015-1094-1)

## Slide 1
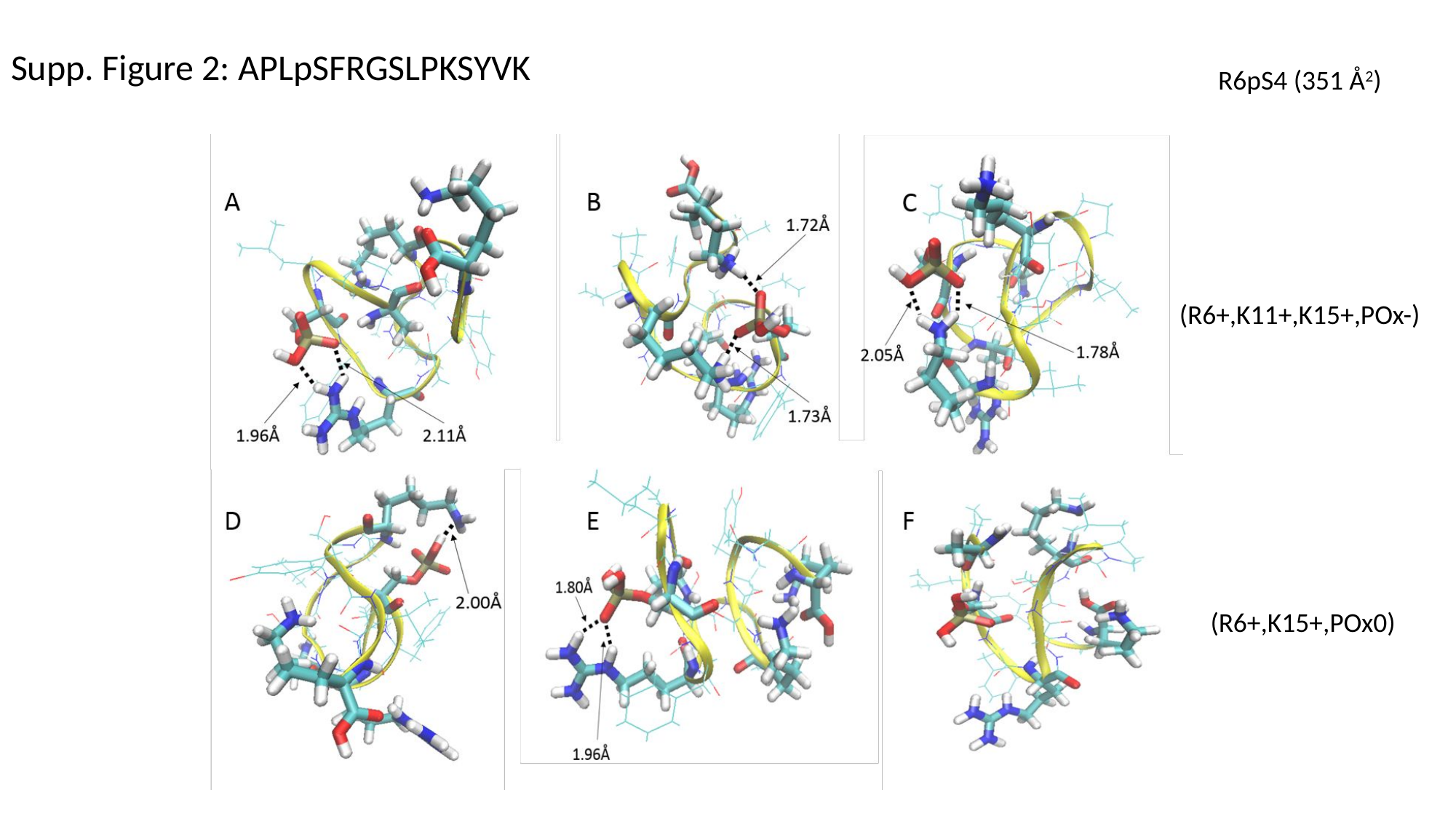

# Supp. Figure 2: APLpSFRGSLPKSYVK
R6pS4 (351 Å2)
(R6+,K11+,K15+,POx-)
(R6+,K15+,POx0)

Supplement: Supplementary file 4 — (PPTX 793 kb) [file 13361_2015_1094_MOESM4_ESM.pptx]

## Slide 1
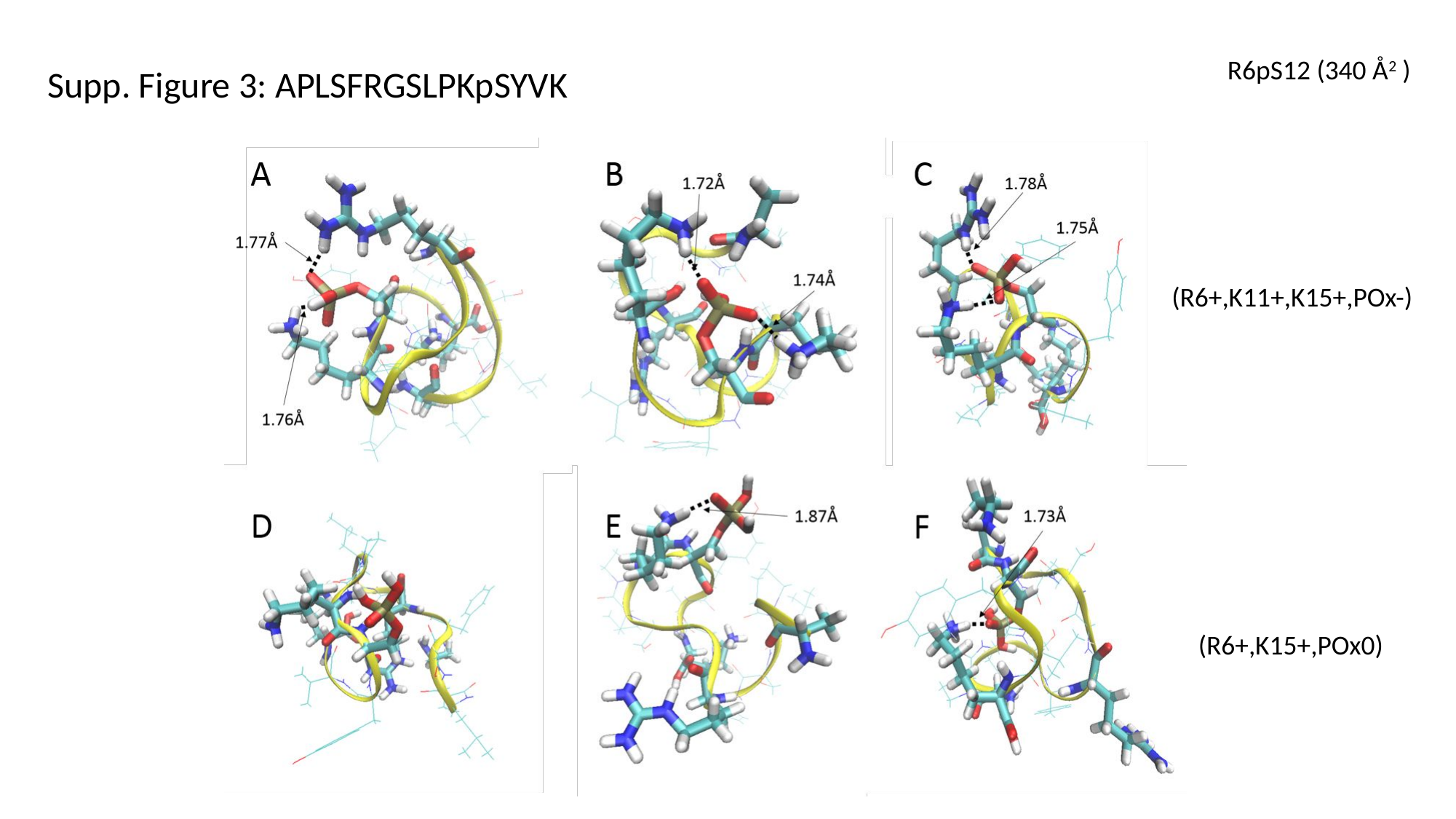

# Supp. Figure 3: APLSFRGSLPKpSYVK
R6pS12 (340 Å2 )
(R6+,K11+,K15+,POx-)
(R6+,K15+,POx0)

Supplement: Supplementary file 5 — (PPTX 752 kb) [file 13361_2015_1094_MOESM5_ESM.pptx]

## Slide 1
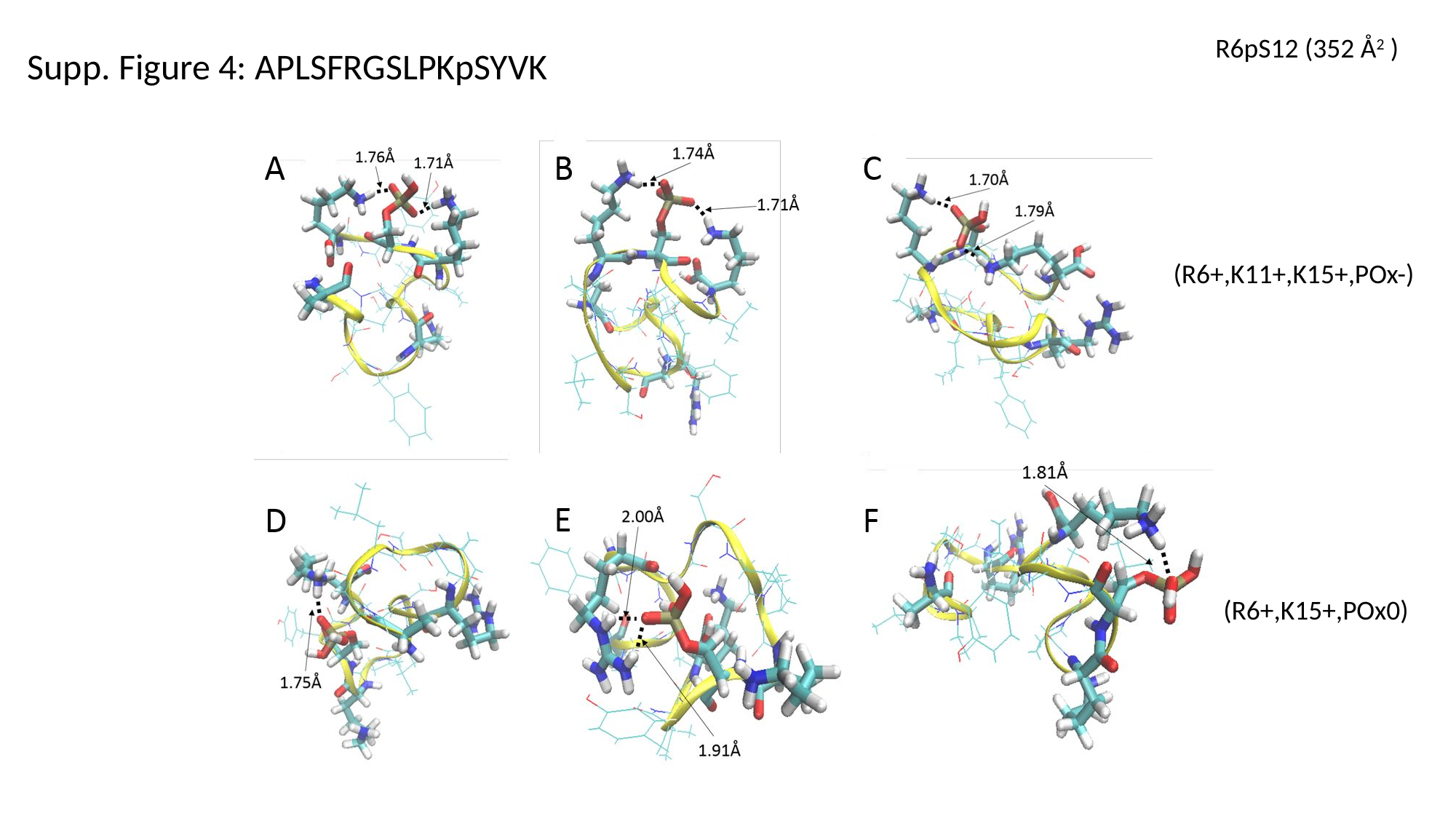

# Supp. Figure 4: APLSFRGSLPKpSYVK
R6pS12 (352 Å2 )
(R6+,K11+,K15+,POx-)
(R6+,K15+,POx0)

Supplement: Supplementary file 6 — (PPTX 749 kb) [file 13361_2015_1094_MOESM6_ESM.pptx]

## Slide 1
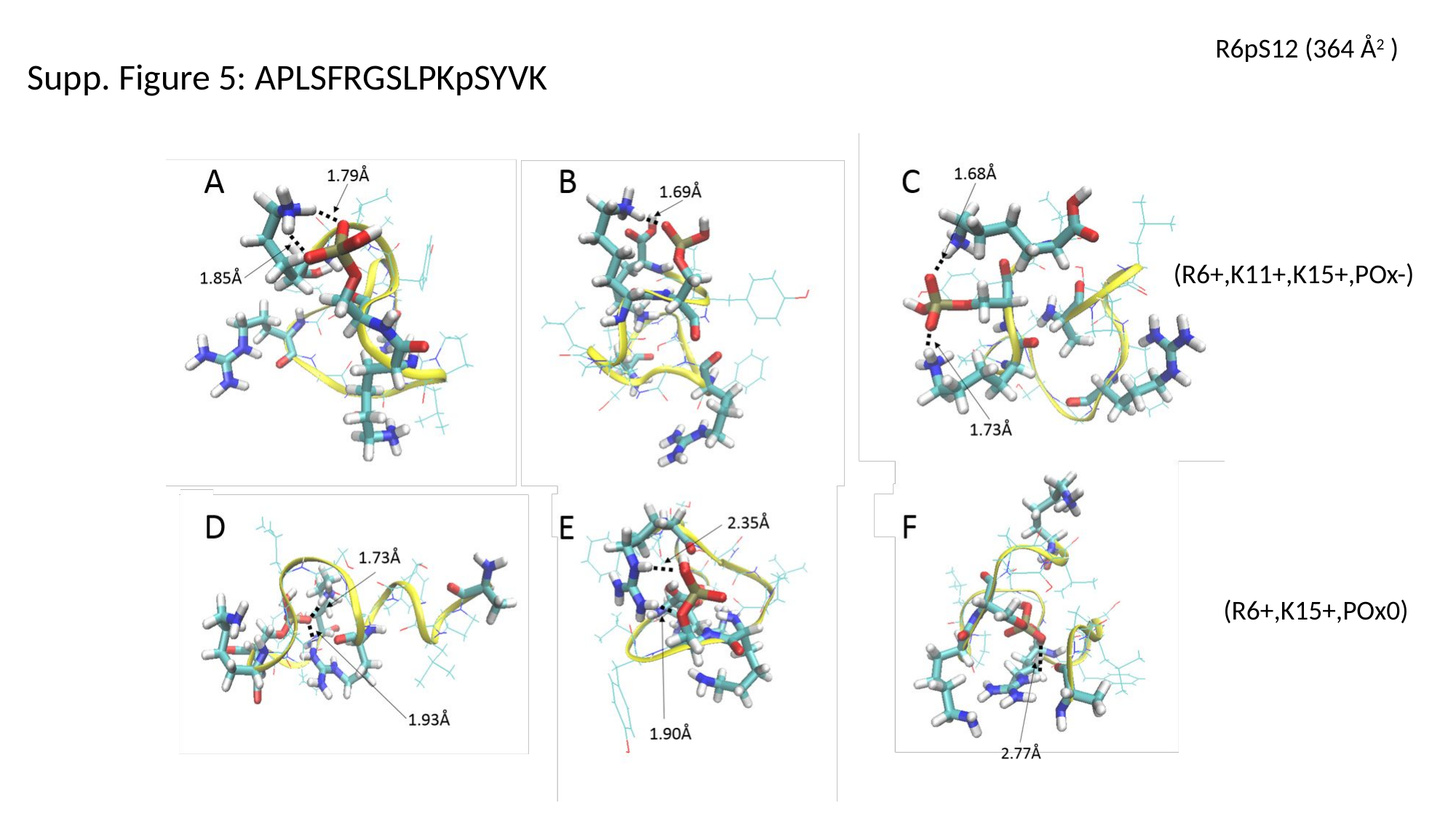

# Supp. Figure 5: APLSFRGSLPKpSYVK
R6pS12 (364 Å2 )
(R6+,K11+,K15+,POx-)
(R6+,K15+,POx0)

Supplement: Supplementary file 7 — (PPTX 759 kb) [file 13361_2015_1094_MOESM7_ESM.pptx]

## Slide 1
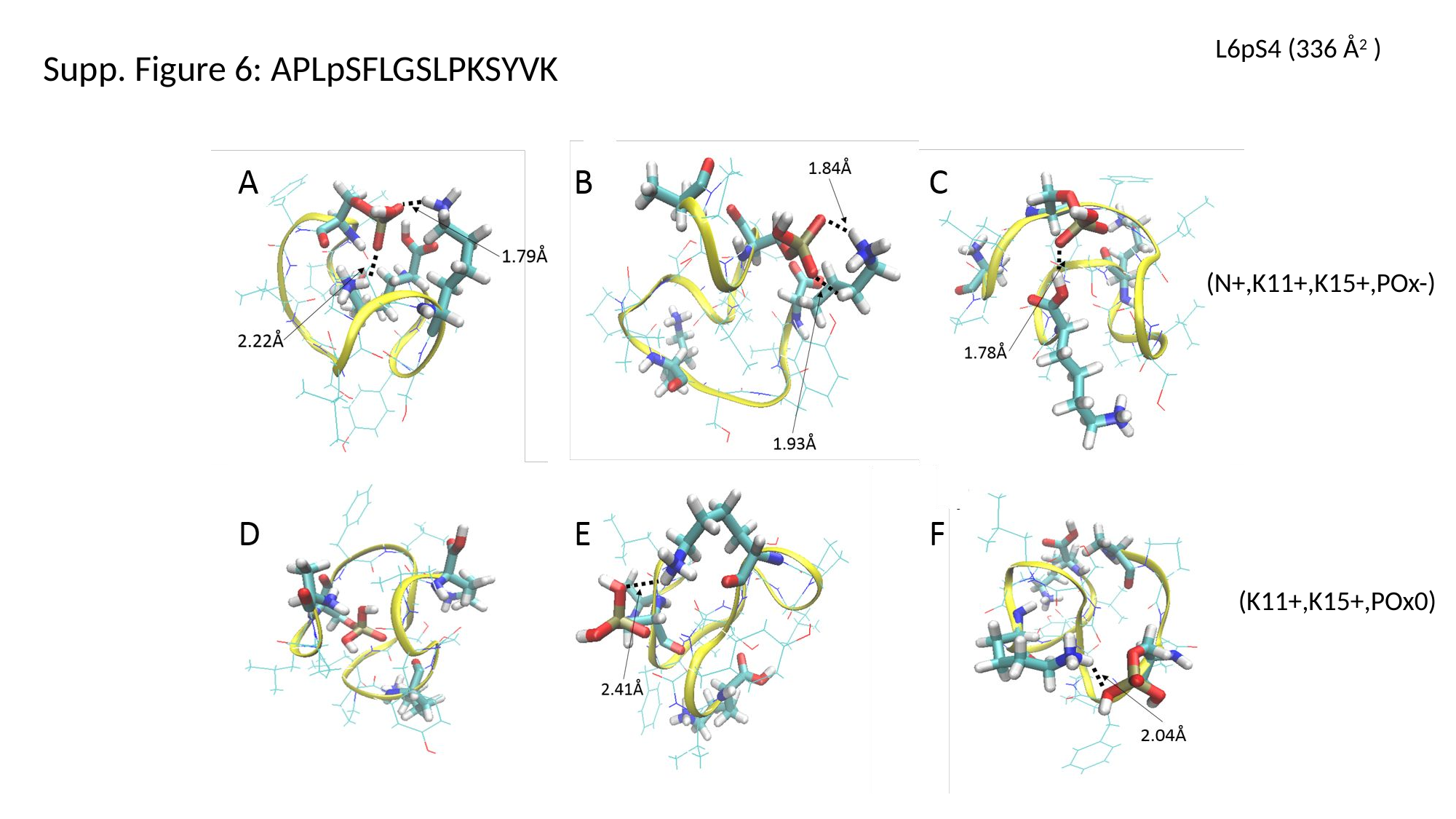

# Supp. Figure 6: APLpSFLGSLPKSYVK
L6pS4 (336 Å2 )
(N+,K11+,K15+,POx-)
(K11+,K15+,POx0)

Supplement: Supplementary file 8 — (PPTX 770 kb) [file 13361_2015_1094_MOESM8_ESM.pptx]

## Slide 1
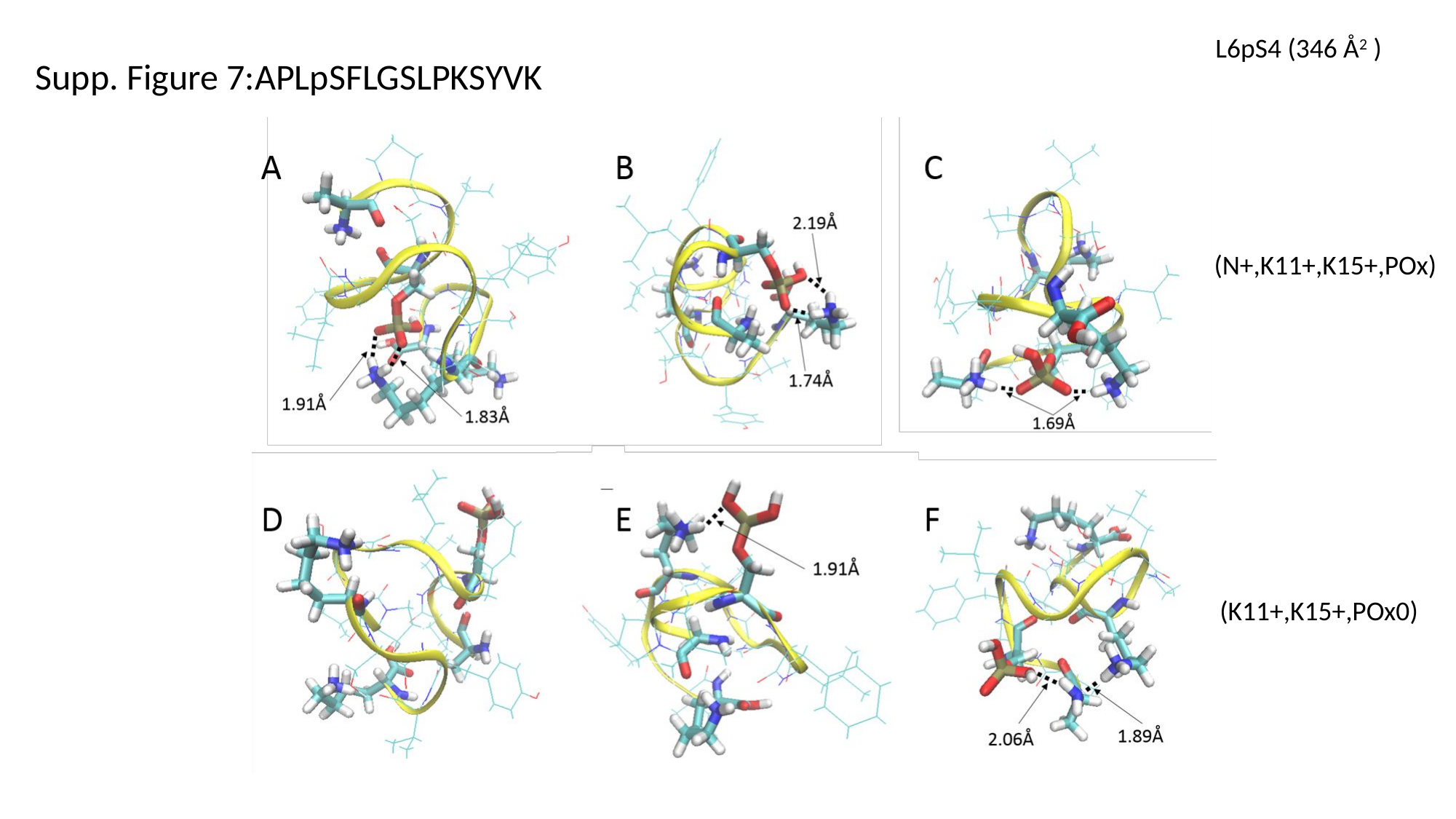

# Supp. Figure 7:APLpSFLGSLPKSYVK
L6pS4 (346 Å2 )
(N+,K11+,K15+,POx)
(K11+,K15+,POx0)

Supplement: Supplementary file 9 — (PPTX 739 kb) [file 13361_2015_1094_MOESM9_ESM.pptx]

## Slide 1
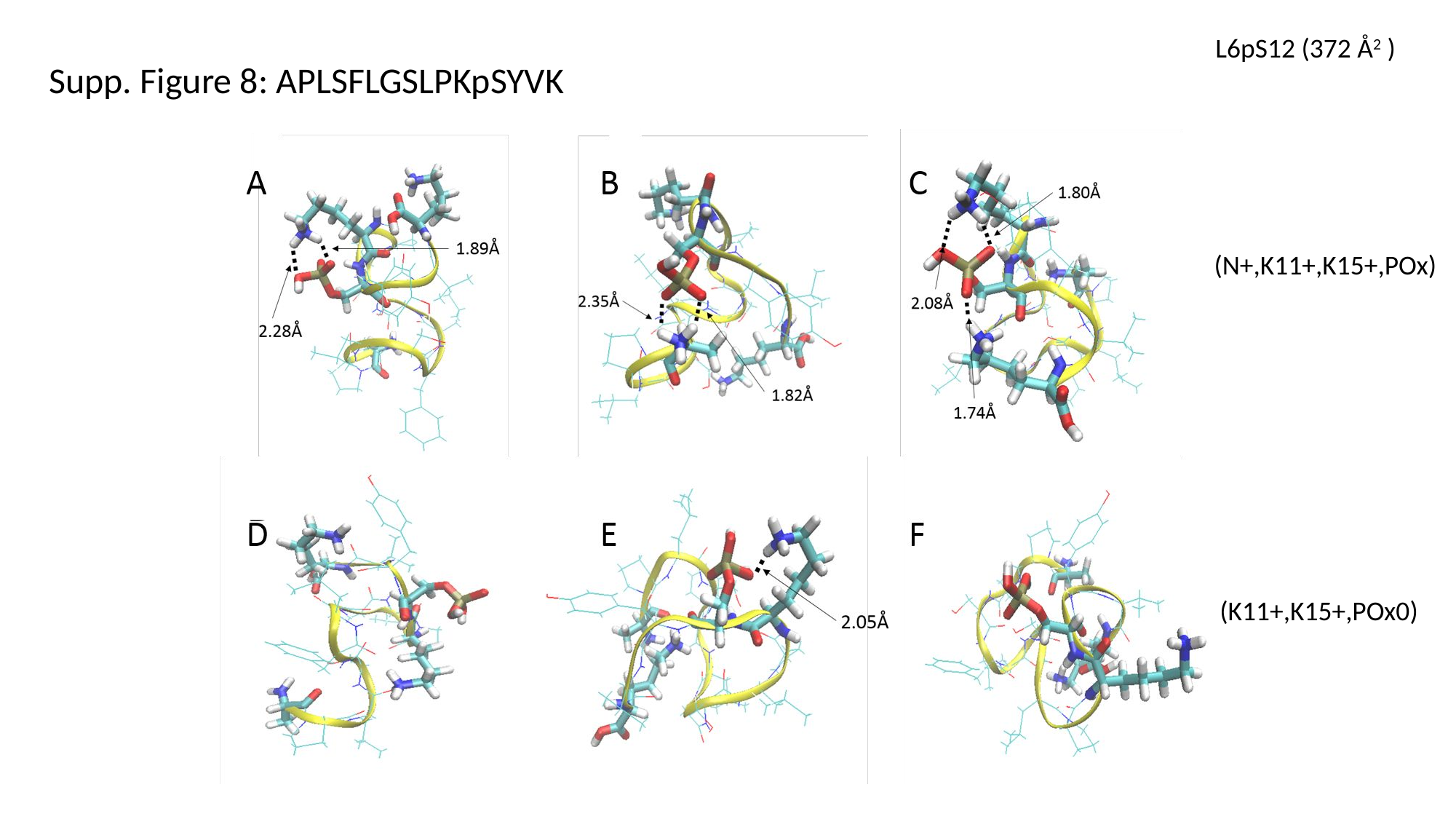

# Supp. Figure 8: APLSFLGSLPKpSYVK
L6pS12 (372 Å2 )
(N+,K11+,K15+,POx)
(K11+,K15+,POx0)

Supplement: Supplementary file 10 — (PPTX 708 kb) [file 13361_2015_1094_MOESM10_ESM.pptx]
